# Evaluation of In Vitro Biofilm Formation of Leptospira Isolates From Human Samples at Four Different Time Frames

**DOI:** 10.7759/cureus.93534

**Published:** 2025-09-30

**Authors:** Yogita Mistry, Summaiya A Mullan, Monika Patel, Pooja Patel

**Affiliations:** 1 Department of Microbiology, Government Medical College, Surat, Surat, IND

**Keywords:** animal husbandry farmer, biofilm formation, biofilm optical density, epidemiology, human isolates, leptospira

## Abstract

Introduction: Biofilm is a group of bacterial cells that are formed through a complex network of intracellular communication, which gives new properties to those organisms, like increased protection and resistance to antimicrobial agents, and decreases the effectiveness of host immune responses. Detection of biofilm and evaluation of biofilm production at different time frames are important parameters for antimicrobial resistance, with no or delayed improvement in patient outcomes even after prolonged antimicrobial therapy.

Material and method: This was an experimental research using 33 human *Leptospira* isolates. Biofilm formation was checked by a quantification method using 96-well polystyrene U-well plates. Biofilm formation was checked on days 5, 7, 14, and 21.

Result: The biofilm optical density (OD) remained relatively low and stable on Day 5 (0.0703) and Day 7 (0.0674), with overlapping 95% Cls, suggesting no significant difference between these two time points. However, a marked increase was observed by Day 14, where the OD rose to 0.1189 (95%CI: 0.0986-0.1391), indicating a significant accumulation of biofilm. This upward trend continued through Day 21, with the highest mean OD recorded at 0.1826 (95% CI: 0.1596-0.2055). The widening confidence intervals on Days 14 and 21 reflect increased variability at later stages.

Conclusion: Human *Leptospira *isolates show potential for biofilm production in a time-dependent manner. In the present study, OD of biofilm remains low and stable on days 5 and 7, with a marked increase in OD seen on Day 14 and much on Day 21.

## Introduction

*Leptospira* is a thin, flexible, elongated, spirally coiled bacillus that comprises two main species, *Leptospira interrogans* and *Leptospira biflexa*, and is classified into more than 300 serogroups and serovars based on lipopolysaccharide antigen. The pathogenic species *L. interrogans* causes leptospirosis, or Weil’s disease, which is a zoonotic disease transmitted to humans mainly by indirect contact with moist soil or water contaminated by infected urine of animals like rats, dogs, cattle, pigs, etc., which also serve as reservoirs for leptospirosis. Direct contact with animals, their parts, placenta, or products of parturition can also cause leptospirosis in humans [1].

Pathogenesis of leptospirosis is mainly due to the septicemic phase, which occurs after entry of bacilli through the conjunctival or oral mucosa or through abraded skin, from where bacilli spill into the bloodstream and disseminate homogeneously to various organs [2]. Due to active motility and release of hyaluronidase, penetration and invasion into tissue occur in association with poor sanitation. It is also responsible for vascular damage and causes vasculitis of small and medium-sized vessels [3,4]. Then comes the immune phase, where antibodies develop and antigen-antibody complexes are deposited in various organs. Mainly, bacilli adhere to the proximal renal tubular brush border and are excreted in urine [5,6].

Biofilm is a group of bacterial cells formed through a complex network of intercellular communication, which confers new properties to organisms, such as increased resistance to antimicrobial agents and decreased effectiveness of host immune response [7]. *Leptospira* with biofilm production can survive for longer durations in water, soil, and rivers, which are important sources for indirect transmission of infection to patients [8,9]. Biofilm production has even been observed within the proximal renal tubules of mammals [10-12], from where continuous shedding of bacteria in urine occurs [13].

Cells within biofilms exhibit increased growth rates, unusual gene expression, enhanced cytotoxic properties, and increased potential for horizontal transfer of resistance genes. All these properties allow multidrug resistance in *Leptospira*, especially to beta-lactam antibiotics, fluoroquinolones, and aminoglycosides [13-15]. Biofilms also provide resistance to biocides used to control *Leptospira* on abiotic surfaces like glassware and biotic surfaces like plants [1,16,17]. Biofilm formation by *Leptospira* may therefore play an important role in their ability to survive in diverse environmental habitats, including hosts. The assessment of biofilm formation potential is fundamental for understanding the epidemiology of the disease and for establishing appropriate prophylaxis and control measures in the local context.

As leptospirosis is common in the South Gujarat region of India, and scattered cases are still present, it is important to understand biofilm formation in *Leptospira* and mature biofilm formation in a time-dependent manner, which can help to understand the pathogenicity of *Leptospira* in patients. Therefore, this study was planned at the Department of Microbiology, Government Medical College, Surat, Gujarat, India, a state-level reference and research center for leptospirosis. The objective of this study was to determine the ability of *Leptospira* isolates from human samples to form biofilm and to perform semi-quantification of biofilm on days 5, 7, 14, and 21 to assess whether mature biofilm formation occurs over time.

## Materials and methods

This experimental study was conducted at the Department of Microbiology, Government Medical College, Surat, which is also a research and reference center for leptospirosis in Western India. The study was approved by the Research Review Committee, Government Medical College, Surat (approval number: GMCS/STU/RRC-2/Approval 8554/25, dated April 15, 2025). The laboratory has 33 leptospiral isolates from human blood samples. For the present study, all 33 isolates were taken to check biofilm formation. These isolates were preserved in semisolid Ellinhausen-McCullough-Johnson-Harris (EMJH) medium at 25 °C. To check biofilm formation, isolates were subcultured on liquid EMJH medium and incubated at 25 °C for five to seven days for exponential growth at log phase. Viability and quality of cultures were evaluated through observation of bacterial population growth and active motility under dark-field microscopy. Lack of contamination was checked by blood culture and Gram staining.

Biofilm formation was done using 96-well polystyrene U-bottom plates and reagents such as 0.1% crystal violet, 30% acetic acid, 4% paraformaldehyde, phosphate-buffered saline (PBS), and 2% sodium acetate. Biofilm formation was checked on days 5, 7, 14, and 21. Four plates were labeled accordingly. Fresh bacterial isolates available in EMJH medium containing approximately 10⁸ *Leptospira*/ml were adjusted to 1 × 10⁶ *Leptospira*/ml using 1% McFarland standard. 150 µl of these cultures were added to the wells. As controls, 150 µl of plain EMJH medium was added to two wells. Other wells were filled with sterile distilled water and sealed with aluminum foil and kept at 30°C [18].

After Day 5, the plate was processed for biofilm detection. Excess medium was aspirated, wells were fixed with paraformaldehyde, stained with crystal violet, washed with PBS, dried, and treated with acetic acid to solubilize the stain. Optical density (OD) was measured at 492 nm using a microplate reader. The same procedure was repeated for days 7, 14, and 21. Data were recorded in Excel (Microsoft Corporation, Redmond, Washington, United States) and analyzed statistically. Mean and standard error of mean (mean±SEM) were calculated. The difference in OD due to biofilm production was assessed using one-way ANOVA and the Tukey test. A p-value <0.05 was considered significant. The ANOVA test was used to evaluate the effect of time on biofilm OD. Mauchly’s test of sphericity for the repeated measures of biofilm OD was used. Bonferroni-adjusted post hoc pairwise comparisons of biofilm OD across the four time points were analyzed. 

## Results

A total of 33 leptospiral isolates were used in the study. Biofolm ODs for different samples were documented at different time frames (Table 1).

**Table 1:** Optical density (OD) obtained on 5th, 7th, 14th and 21st days at 492 nm filter

Serial No	Biofilm OD of 5th day	Biofilm OD of 7th day	Biofilm OD of 14th day	Biofilm OD of 21st day
1	0.08	0.05	0.09	0.22
2	0.06	0.09	0.09	0.16
3	0.07	0.09	0.08	0.21
4	0.06	0.07	0.34	0.24
5	0.05	0.06	0.1	0.2
6	0.06	0.07	0.14	0.17
7	0.04	0.05	0.13	0.15
8	0.05	0.07	0.13	0.12
9	0.06	0.06	0.08	0.14
10	0.07	0.07	0.08	0.19
11	0.08	0.09	0.09	0.17
12	0.14	0.05	0.09	0.2
13	0.05	0.07	0.1	0.2
14	0.06	0.04	0.13	0.16
15	0.06	0.09	0.13	0.13
16	0.04	0.07	0.11	0.16
17	0.07	0.06	0.09	0.13
18	0.1	0.1	0.1	0.14
19	0.1	0.07	0.08	0.16
20	0.08	0.08	0.09	0.17
21	0.07	0.07	0.32	0.19
22	0.06	0.06	0.12	0.24
23	0.09	0.09	0.15	0.48
24	0.05	0.04	0.12	0.13
25	0.1	0.07	0.08	0.15
26	0.07	0.05	0.08	0.16
27	0.08	0.07	0.09	0.25
28	0.06	0.06	0.1	0.13
29	0.07	0.09	0.2	0.15
30	0.05	0.06	0.13	0.3
31	0.08	0.07	0.13	0.23
32	0.05	0.06	0.12	0.15
33	0.09	0.06	0.08	0.12
Control-1	0.08	0.05	0.08	0.13
Control-2	0.08	0.06	0.09	0.16

Table 2 presents the estimated marginal means of biofilm OD measured at four different time points. The biofilm OD remained relatively low and stable on Day 5 (0.0703) and Day 7 (0.0674), with overlapping 95% CIs, suggesting no significant difference between these two time points. However, a marked increase was observed by Day 14, where the OD rose to 0.1189 (95% CI: 0.0986-0.1391), indicating a significant accumulation of biofilm. This upward trend continued through Day 21, with the highest mean OD recorded at 0.1826 (95%CI: 0.1596-0.2055). The widening CIs on Days 14 and 21 reflect increased variability at later stages. Overall, the data demonstrate a time-dependent increase in biofilm formation, particularly after Day 7, with statistically and biologically meaningful elevations in optical density. 

**Table 2:** Estimated marginal means of biofilm optical density over time

Biofilm OD	Mean	SE	95% CI	
			Lower	Upper
Day 5	0.0703	0.00344	0.0633	0.0773
Day 7	0.0674	0.00257	0.0622	0.0727
Day 14	0.1189	0.00997	0.0986	0.1391
Day 21	0.1826	0.01130	0.1596	0.2055

**Figure 1:**
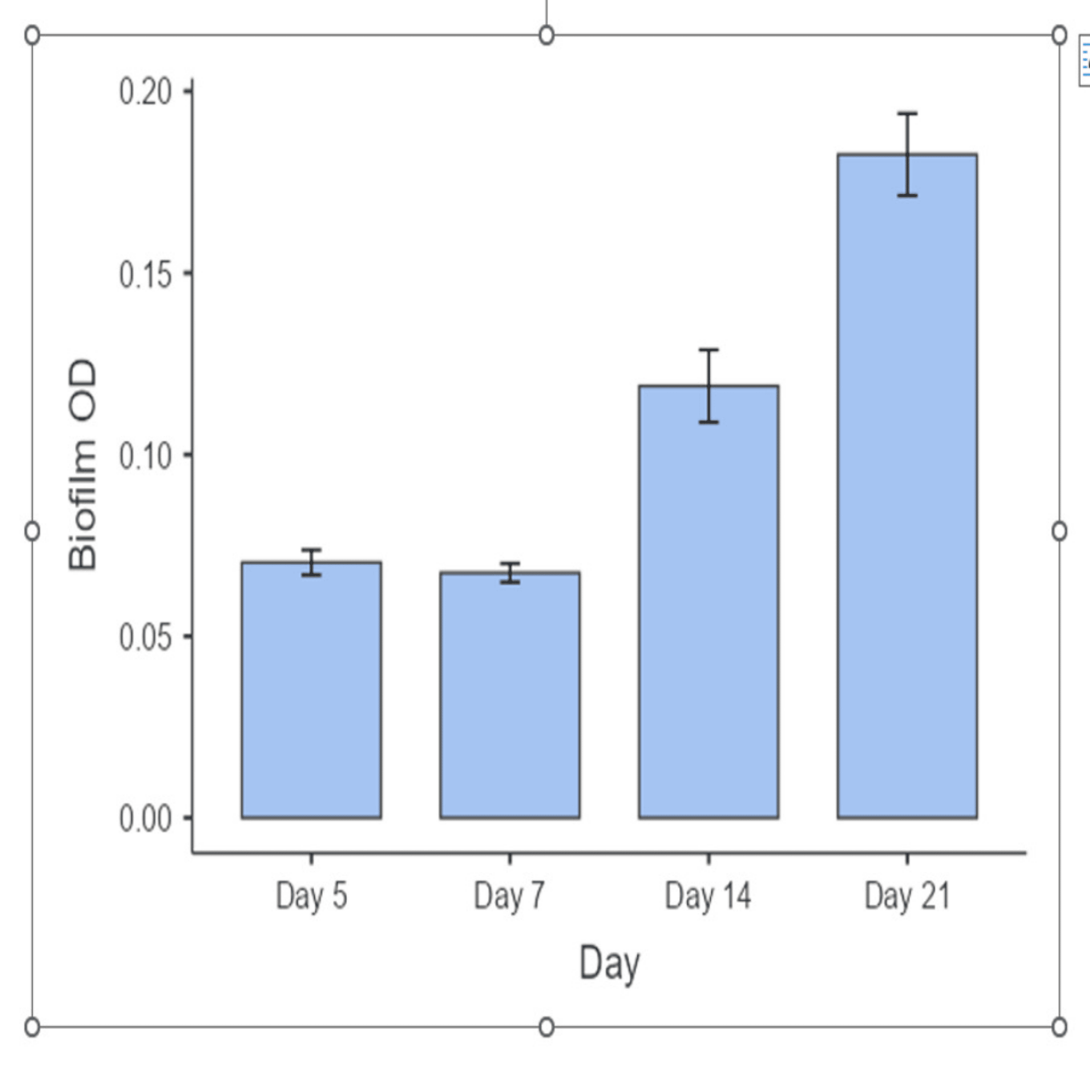
Temporal progression of biofilm formation measured by optical density (OD)

Table 3 summarizes the results of a repeated measures ANOVA evaluating the effect of time on biofilm OD. The analysis revealed a statistically significant effect of time on biofilm OD (F(3,102) = 52.0, p < .001), indicating that the OD values varied significantly across the different time points. The generalized eta squared (η²G = 0.511), eta squared (η² = 0.511), and partial eta squared (η²p = 0.605) values all suggest a large effect size, with over 60% of the variance in biofilm OD attributable to the effect of time when controlling for individual differences. This confirms a strong and meaningful temporal trend in biofilm development. In Figure 1, the temporal progression of biofilm formation measured by OD has been shown. 

**Table 3:** Repeated measures ANOVA summary for biofilm optical density (OD) across time points The effect is highly significant (F(3,102) = 52.0, p < .001). Effect sizes: η²G = 0.511 → large effect; η² = 0.511 → large effect; η²p = 0.605 → very large effect. This means that biofilm OD explains around 51–60% of the variance depending on which effect size metric  report. That’s a very strong effect.

	Sum of Squares	DF	Mean Square	F	p	η²_G_	η²	η²_p_
Biofilm OD	0.306	3	0.10190	52.0	< .001>	0.511	0.511	0.605
Residual	0.200	102	0.00196					

The results of Mauchly’s test of sphericity for the repeated measures of biofilm OD. The test indicated a significant violation of the sphericity assumption (Mauchly’s W = 0.260, p < .001), which suggests that the variances of the differences between time points are not equal. Due to this violation, corrections to the degrees of freedom are necessary for Figure 1. Temporal progression of biofilm formation measured by OD ensures the validity of the ANOVA results. The Greenhouse-Geisser epsilon (ε = 0.714) and the Huynh-Feldt epsilon (ε = 0.764) values are both less than 1, supporting the need for adjusted F-ratios. These corrections were therefore applied in the repeated measures ANOVA to provide a more accurate assessment of the time effect on biofilm OD.

Table 4 presents the results of Bonferroni-adjusted post hoc pairwise comparisons of biofilm OD across the four time points. No statistically significant difference was observed between Day 1 and Day 2 (mean difference = 0.00286, p = 1.000), indicating that biofilm formation remained stable in the early phase. However, significant increases in OD were observed from Day 3 onward. Comparisons between Day 1 and Day 3 (mean difference = -0.04857, p < .001), and between Day 1 and Day 4 (mean difference = -0.11229, p < .001) were both statistically significant, indicating a substantial increase in biofilm formation over time. Similarly, Day 2 showed significantly lower OD compared to both Day 3 and Day 4 (p < .001). The comparison between Day 3 and Day 4 also revealed a significant increase in OD (mean difference = -0.06371, p < .001). These findings confirm a progressive and statistically significant accumulation of biofilm from Day 3 onward.

**Table 4:** Pairwise comparisons of biofilm optical density (OD) using Bonferroni correction Biofilm OD values did not change significantly between Day 1 and Day 2, but from Day 3 onwards there was a progressive and statistically significant reduction, with the lowest OD at Day 4.

Days			Mean Difference in Biofilm OD	SE	df	t	p_bonferroni_
Day 1	-	Day 2	0.00286	0.00399	34.0	0.717	1.000
	-	Day 3	-0.04857	0.01120	34.0	-4.337	< .001>
	-	Day 4	-0.11229	0.01138	34.0	-9.870	< .001>
Day 2	-	Day 3	-0.05143	0.01002	34.0	-5.134	< .001>
	-	Day 4	-0.11514	0.01109	34.0	-10.385	< .001>
Day 3	-	Day 4	-0.06371	0.01332	34.0	-4.785	< .001>

## Discussion

Biofilm formation by *Leptospira* may play an important role in their ability to survive in diverse environmental habitats, including the host. Assessment of biofilm production and its analysis at different time frames helps us understand the importance of early identification of infection and early treatment. As biofilm becomes stronger on Day 14 and Day 21, if patients are not treated early, there is a higher chance of treatment failure or poor response due to biofilm development.

In the present study, which was conducted with 33 human isolates, biofilm formation was evaluated in U-bottom well plates over time. Biofilm formation was analyzed on days 5, 7, 14, and 21. The OD values of biofilm remained low and stable on days 5 and 7, with a marked increase on Day 14 and an even higher increase on Day 21. This suggests a time-dependent increase in biofilm formation. Greater biofilm production was observed with isolates 3, 4, 22, and 31 on Day 21, suggesting their possible adaptability in in vitro maintenance due to individual strain characteristics. Biofilms have been shown to influence *L. interrogans* environmental survival, infection pathogenesis, and persistence in reservoir hosts. This suggests the potential of biofilm formation even on other surfaces, including environmental matrices such as water collections, rivers, soil, abiotic surfaces, and within the proximal renal tubules of the host [10-13]. Our results are in agreement with the studies by Rezende Mires de Carvalho et al. [18] and Thibeaux et al. [19]. Analysis of biofilm formation in animal isolates or environmental samples is also important, as it helps evaluate the efficacy of currently used rodent control measures or biocides [20].

Limitations

Isolates that were tested here were preserved in the Leptospirosis Reference Labs. We were not able to retrieve patients' detailed history and their clinical outcomes. So we could not address whether isolates having high OD in biofilm have poor clinical outcomes, high morbidity, etc., or not. In the present study, animal isolates, which are also important to study to understand epidemiology and help in modifying treatment and preventive measures, were not studied. These are useful at the field level to control *Leptospira* in the environment and in the host. 

## Conclusions

Human *Leptospira* isolates show potential for biofilm formation in a time-dependent manner. In the present study, the OD of biofilm remains low and stable on days 5 and 7, with a marked increase in OD seen on Day 14 and much on Day 21.
